# Linkage and association of myocilin (*MYOC*) polymorphisms with high myopia in a Chinese population

**Published:** 2007-04-04

**Authors:** Wing Chun Tang, Shea Ping Yip, Ka Kin Lo, Po Wah Ng, Pik Shan Choi, Sau Yin Lee, Maurice K.H. Yap

**Affiliations:** 1 School of Optometry, The Hong Kong Polytechnic University, Hung Hom, Kowloon, Hong Kong SAR, China; 2 Department of Health Technology and Informatics, The Hong Kong Polytechnic University, Hung Hom, Kowloon, Hong Kong SAR, China

## Abstract

**Purpose:**

To test the association between myocilin gene (*MYOC*) polymorphisms and high myopia in Hong Kong Chinese by using family-based association study.

**Methods:**

A total of 162 Chinese nuclear families, consisting of 557 members, were recruited from an optometry clinic. Each family had two parents and at least one offspring with high myopia (defined as -6.00D or less for both eyes). All offspring were healthy with no clinical evidence of syndromic disease and other ocular abnormality. Genotyping was performed for two *MYOC* microsatellites (NGA17 and NGA19) and five tag single nucleotide polymorphisms (SNPs) spreading across the gene. The genotype data were analyzed with Family-Based Association Test (FBAT) software to check linkage and association between the genetic markers and myopia, and with GenAssoc to generate case and pseudocontrol subjects for investigating main effects of genetic markers and calculating the genotype relative risks (GRR).

**Results:**

FBAT analysis showed linkage and association with high myopia for two microsatellites and two SNPs under one to three genetic models after correction for multiple comparisons by false discovery rate. NGA17 at the promoter was significant under an additive model (p=0.0084), while NGA19 at the 3' flanking region showed significant results under both additive (p=0.0172) and dominant (p=0.0053) models. SNP rs2421853 (C>T) exhibited both linkage and association under additive (p=0.0009) and dominant/recessive (p=0.0041) models. SNP rs235858 (T>C) was also significant under additive (p=4.0E-6) and dominant/recessive (p=2.5E-5) models. Both SNPs were downstream of NGA19 at the 3' flanking region. Positive results for these SNPs were novel findings. A stepwise conditional logistic regression analysis of the case-pseudocontrol dataset generated by GenAssoc from the families showed that both SNPs could separately account for the association of NGA17 or NGA19, and that both SNPs contributed separate main effects to high myopia. For rs2421853 and with C/C as the reference genotype, the GRR increased from 1.678 for G/A to 2.738 for A/A (p=9.0E-4, global Wald test). For rs235858 and with G/G as the reference, the GRR increased 2.083 for G/A to 3.931 for A/A (p=2.0E-2, global Wald test). GRR estimates thus suggested an additive model for both SNPs, which was consistent with the finding that, of the three models tested, the additive model gave the lowest p values in FBAT analysis.

**Conclusions:**

Linkage and association was shown between the *MYOC* polymorphisms and high myopia in our family-based association study. The SNP rs235858 at the 3' flanking region showed the highest degree of confidence for association.

## Introduction

Myopia is a common eye problem worldwide and is much more prevalent in Asian populations than in Caucasian populations [1-4]. A high degree of myopia increases the risk of developing sight-threatening ocular pathology, such as retinal degeneration and glaucoma [5,6]. Thus, the impact of myopia on public health care and economy is enormous.

Myopia is a complex trait [7-10], although some cases of high myopia show patterns of Mendelian inheritance [11-20]. Complex traits are determined by both genetic and environmental factors and possibly their interactions. They may run in families but they do not always show typical patterns of Mendelian inheritance [21,22]. Identification of susceptibility genes for myopia will shed light on the underlying genetic mechanisms. Such information is important for the design of new treatment to prevent or slow down myopia development. Several myopia loci have been identified by parametric linkage analysis based on the assumption of an autosomal-dominant mode of inheritance [11-18]. A twins study also demonstrated significant linkage of myopia at chromosome 11p13 by nonparametric linkage analysis [23]. Linkage analysis has been successful in identifying genes of large effect size in monogenic diseases showing typical Mendelian inheritance patterns, but has limited power in detecting small genetic effects in complex traits [21,22,24]. True linkage will also be missed should a wrong genetic model be assumed in parametric linkage analysis [25]. A genetic association study provides an alternative that is more powerful in detecting small genetic effects in complex traits [21,22,24].

The myocilin gene (*MYOC*; OMIM 601652), which is located on chromosome 1q24-q25, contains three exons and encodes a structural protein of 504 amino acids called myocilin. This protein was originally known as the trabecular meshwork-induced glucocorticoid response protein (TIGR) [26-28]. Mutations in *MYOC* have been identified as the cause of primary open-angle glaucoma and the risk factors of different types of glaucoma [29,30]. *MYOC* is expressed in many ocular tissues, including the trabecular meshwork, ciliary bodies, sclera, and choroids [31]. There is an increased frequency of open-angle glaucoma in myopes as well as an increased prevalence of myopia in patients with glaucoma or ocular hypertension [32-34]. Although it is still not clear whether increased intraocular pressure plays a role in the weakening of sclera and the ocular enlargement in myopia, there is evidence of higher intraocular pressure in myopic eyes compared to emmetropic eyes [35]. Thus, we hypothesize that polymorphisms in and around the *MYOC* gene may play a role in myopia susceptibility.

**Figure 1:**
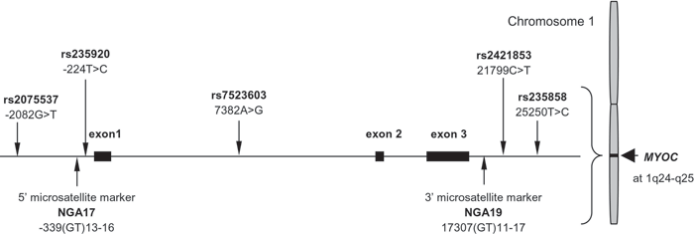
The structure of the *MYOC* gene and the locations of genetic markers tested in the study. Microsatellites were named according to The GDB Human Genome Database and single nucleotide polymorphisms with rs numbers. Their positions are indicated according to the recommendations of the Human Genome Variation Society (nomenclature for the description of sequence variations).

Two polymorphic microsatellites are on the *MYOC* locus, and both are GT repeats: NGA17 at the promoter and NGA19 at the 3' flanking region (Figure 1) [26,27,31]. Three small studies tested the association between *MYOC* and myopia but results conflicted [36-38]. The present study aimed to clarify the relationship between the *MYOC* microsatellites and high myopia using a large number of Chinese families living in Hong Kong. The relationship was further delineated by investigating additional tag single nucleotide polymorphisms (SNPs) spreading across the *MYOC* gene (Figure 1). A family-based association study approach was used to avoid false positive results due to population stratification [39].

## Methods

### Subjects and DNA samples

Unrelated nuclear families of Chinese descent were recruited from the optometry clinic of Hong Kong Polytechnic University. Each family consisted of at least one myopic offspring who had a refractive error of -6.00D or less (spherical power) for both eyes, and their parents. Each subject received a comprehensive eye examination, including visual acuity, refraction, slit lamp, and dilated fundus examination. Objective refraction of each subject was taken using open field autorefractor (Shin-Nippon SRW-5000, Tokyo, Japan) after the subject was given one to two drops of 1% tropicamide per eye. Central corneal curvature was measured using autokeratometry (Canon RK-5 Auto Ref-keratometer, Canon, Inc., Tokyo, Japan). Refraction and corneal curvature measurement were carried out prior to axial length measurement. Axial length was measured using A-Scan ultrasound (Advent A/B System; Mentor, Santa Barbara, CA). Before the ultrasound measurement, one drop of 0.4% benoxinate hydrochloride was instilled in each eye to produce anesthesia. Myopic offspring were excluded from the study if they showed obvious signs of ocular disease or other inherited disease associated with myopia (e.g., Stickler syndrome and Marfan syndrome). The age of first spectacle wear for myopia was recorded and used as a surrogate for the approximate myopia onset age.

Venous blood samples were collected, and DNA was extracted from the leukocytes using a modified salt precipitation method. This study was approved by the Human Subjects Ethics Subcommittee of the Hong Kong Polytechnic University, and adhered to the tenets of the Declaration of Helsinki. Written informed consent was obtained from every subject.

### Microsatellite genotyping

The *Homo sapiens* chromosome 1 genomic contig NT_004487 was used as the reference genomic DNA sequences. Primers were designed to amplify the sequences flanking the two microsatellites by polymerase chain reaction (PCR). The forward primers (Myocpm-F and Myoc3pm-F) for both reactions were labeled with fluorescein at the 5' end (Table 1). The reaction mixture (15 μl) contained 1X Gold Buffer (15 mM Tris-HCl, 50 mM KCl, pH 8.0), 0.2 mM of each dNTP, 1.5 mM MgCl_2_, 0.3 μM of each primer for NGA17 (or 0.5 μM for NGA19; Table 1), 1 U AmpliTaq Gold DNA polymerase (Applied Biosystems, Foster City, CA) and 20 ng genomic DNA. Touchdown PCR was used to avoid excessive optimization of the reaction conditions [40]. PCR amplification consisted of initial denaturation for 5 min at 95 °C, 8 touchdown cycles, 30 main cycles, and final extension for 7 min at 72 °C. Both touchdown and main cycles consisted of 30 s at 95 °C, 45 s at the annealing temperature and 45 s at 72 °C. The annealing temperature of the main cycles was 53 °C for NGA17 and 57 °C for NGA19, and the initial annealing temperature for the touchdown cycles was 7 °C above this with 1 °C reduction for each successive touch-down cycle. As the two PCR fragments were sufficiently different in size (about 330 bp for NGA17 and about 145 bp for NGA19), the two PCR products separately amplified from the same individual were mixed and genotyped in the same injection. The PCR products were sized on the ABI PRISM 310 Genetic Analyzer using Genescan software (Applied Biosystems) together with GeneScan-500 (TAMRA) size standard (Applied Biosystems) according to the manufacturer's instructions, and the genotypes were called manually.

**Table 1:** Genotyping of *MYOC* microsatellites and single nucleotide polymorphisms.

**Marker**	**Marker type/assay method**	**Primer/probe/assay ID**	**5'-3' Sequence/allele (probe signal)**
NGA17	MS/Fragment analysis	Myocpm-F	FLU-GGCTGTTATTTTTCTCTGT
		Myocpm-R	TGCCAGCAAGATTCTTAGAA
NGA19	MS/Fragment analysis	Myoc3pm-F	FLU-GTTGGGAGATGTGATTGCAG
		Myoc3pm-R	AGATGGAGGTGGGAAAGTGT
rs7523603	SNP/TaqMan Assay-by-Design	Myocil-F	CGGACCCAGAGCGAAGTT
		Myocil-R	AGGGCTGTGGAAAGGTTATGG
		A allele-specific probe	VIC-CTGTGAGGTCAC[A]GAAG-MGB
		G allele-specific probe	FAM-TGTGAGGTCAC[G]GAAG-MGB
rs2075537	SNP/TaqMan Assay-on-Demand	C_27532255_10	T (VIC) and G (FAM)
rs235920	SNP/TaqMan Assay-on-Demand	C_558534_10	T (VIC) and C (FAM)
rs2421853	SNP/TaqMan Assay-on-Demand	C_11335131_10	A (VIC) and G (FAM)
rs235858	SNP/TaqMan Assay-on-Demand	C_2964922_10	A (VIC) and G (FAM)

Forward primers that amplified microsatellites (MS) were labeled with fluorescein (FLU). Four single nucleotide polymorphisms (SNPs) were genotyped by assay-on-demand TaqMan. Only assay identifications and allele-specific probe signals were provided by the manufacturer. All allele-specific probes were labeled with a reporter dye (either VIC or FAM) at the 5' end, and a quencher and a minor groove-binder (MGB) at the 3' end. Bases in square brackets indicate the positions of the bases defining the alleles of the SNPs that were to be detected by the probes.

### Single nucleotide polymorphism genotyping

Using the HapMap database, we selected five tag SNPs (Figure 1) from the *MYOC* gene with the selection criteria of r^2^>0.8 and minor allele frequency >0.15 for the Han Chinese population. The SNPs were genotyped with TaqMan SNP Genotyping Assays (Applied Biosystems; Table 1). TaqMan assays consist of unlabeled PCR primers and TaqMan MGB probes (separately labeled with FAM and VIC). The reaction mixture (10 μl) contained 5 μl of 4X TaqMan Universal PCR Master Mix (Applied Biosystems), 0.5 μl of 20X TaqMan SNP Genotyping Assay Mix (Applied Biosystems) and 4.5 μl of 10-20 ng genomic DNA. The PCR cycling conditions included an initial denaturation for 10 min at 95 °C plus 50 cycles of denaturation for 15 s at 92 °C, annealing and extension for 1 min at 60 °C. All PCRs were performed in 96-well plates with either a GeneAmp PCR System 9700 thermocycler or a 7500 Real-Time System (both from Applied Biosystems). The plates, containing amplified PCR products, were read with a 7500 Real-Time System and then analyzed with the Sequence Detection System software (version 1.2.2, Applied Biosystems) for allelic discrimination.

### Statistical analysis

Genotype distribution in parents was assessed for departures from expectations based on Hardy Weinberg equilibrium by the χ^2^ test. Two linkage disequilibrium (LD) measures were calculated, namely, Lewontin's D' and the correlation coefficient r^2^ [41]. LD involving at least one microsatellite was first calculated for each allele-pair, and the locus-pair LD statistic then calculated as a weighted mean of the absolute allele-pair LD measures [42]. Calculation was done using the software PowerMarker (version 3.23).

Tests of individual markers for association with myopia were performed with the Family-based Association Test package (FBAT, version 1.7.2) [43,44]. Briefly, FBAT uses a generalized score test to perform a variety of tests similar in spirit to a transmission/disequilibrium test [39]. FBAT is not susceptible to biases due to population admixture, misspecification of the trait distribution, as well as selection based on trait [43,44]. Since there was no evidence for linkage of myopia susceptibility locus to the *MYOC* locus or chromosomal region 1q24-25, the null hypothesis in this case was "no linkage and no association between the marker locus and any trait-influencing locus." The alternate hypothesis was that "there was both linkage and association." FBAT permits multiple affected offspring per family, handles multi-allelic markers, and allows different genetic models to be tested. The effect of a risk allele does the following: (1) It increases with its copy number in an additive model; (2) it is the same for one or two copies in a dominant model; and (3) it is demonstrated only with two copies of the risk allele in a recessive model.

The multiple testing issues for the alleles of a given marker were solved by the global statistic for each marker tested under any given genetic model. For the global statistics, there were seven genetic markers (two microsatellites and five SNPs), each tested under three different genetic models. For biallelic SNP markers, dominant and recessive models give reciprocal results and thus are equivalent to one test for the purpose of accounting for multiple testing [45]. Thus, there were 16 tests of global association. The more powerful false discovery rate (FDR) [46], instead of the conventional Bonferroni procedure, was used to control for multiple hypothesis testing. After adjustment for multiple testing and with an FDR level of 0.05, the cut-off for significant global association was 0.0219.

A matched case-control dataset was generated with each affected (myopic) offspring compared to three possible pseudocontrols created from the untransmitted parental allele [47,48]. For NGA17 and NGA19, the case-pseudocontrol dataset was generated after grouping all non-5 alleles as one allele; this served to group less common alleles together. This case-pseudocontrol dataset was analyzed by a stepwise conditional logistic regression (CLR) procedure [47] to determine whether one or more markers could be identified as the primary associated marker(s) among those found positive by the FBAT analysis. Briefly, markers were entered into the CLR equation in a stepwise forward manner to investigate whether a marker was still significantly associated with high myopia after taking into consideration the main effects of one or more other markers. When markers were found to be primarily associated with high myopia, CLR was again used to calculate the effect size of the marker genotype on the disease risk as the genotype relative risk (GRR) and the corresponding 95% confidence intervals. Robust variance estimates account for nonindependence of observations within nuclear families with multiple affected offspring and were thus used in the CLR analysis. Accordingly, significance was assessed by the Wald χ^2^ test. CLR was performed with the GenAssoc package and executed within the software STATA (version 8.2; Stata Corporation, College Station, TX).

## Results

### Subjects

In total, 557 individuals were recruited for this study from 162 nuclear families comprising 324 parents and 233 myopic offspring. Both parents were available in every nuclear family. For 21 families, one of the parents had high myopia (-6.00D or less for both eyes) whereas in only one family both parents had high myopia. The recruited families each had one (95 families), two (63 families). or three (4 families) myopic offspring. The mean age of the myopic offspring was 24.9 years (SD=7.5 years) and there were more female offspring (65.7%) than male. A summary of the ocular data is shown in Table 2. The mean spherical power of myopic offspring was -8.38D (SD=-1.90D) and -8.27D (SD=-1.89D) for right and left eyes, respectively. The age range of myopia onset for most offspring (88%) was 6-15 years.

**Table 2:** Summary of ocular data of myopic offspring.

**Ocular parameter (unit)**	**Right eye**	**Left eye**
Spherical power (D)	-8.38±1.90	-8.27±1.89
Equivalent spherical power (D)	-9.06±2.01	-9.10±2.00
Astigmatism (D)	-1.35±0.88	-1.67±0.96
Corneal cylindrical power (D)	-1.54±0.73	-1.65±0.79
Axial length (mm)	26.88±1.05	26.86±1.14
Anterior chamber depth (mm)	3.57±0.34	3.54±0.35

This study involved a total of 233 myopic offspring. The table shows the distribution of refractive errors and size-related measurements of both eyes in the myopic offspring. All ocular data are expressed as mean±standard deviation. D represents diopters.

### Allele frequencies and LD for genetic markers

The distribution of allele frequencies in the parents is shown in Table 3. The genotypes of all seven markers were in Hardy-Weinberg proportions (p>0.05). For microsatellites, four alleles were found for NGA17 and six alleles for NGA19. For the sake of consistency, alleles with 11 to 17 GT repeats were designated as alleles 1 to 7, respectively although alleles 1, 2, and 7 were not found for NGA17 and allele 2 was not identified for NGA19 in this study. The most common allele had 13 GT repeats (allele 3, frequency=0.5015) for NGA17, and 15 GT repeats (allele 5, frequency=0.7114) for NGA19. For SNPs, the major allele was designated as allele 1 and the minor allele as allele 2 (Table 3). The minor allele frequency was lowest for rs7523603 (0.2438 for G allele) and highest for rs235920 (0.4645 for C allele).

**Table 3:** *MYOC* polymorphisms: summary of genetics data for parents and tests of association by family-based association testing under different genetic models in 162 nuclear families.

**Marker**	**rs2075537**			**NGA17**						**rs235920**			**rs7523603**	
Allele	1 (G)	2 (T)		3 (13)	4 (14)		5 (15)		6 (16)	1 (T)		2 (C)	1 (A)	2 (G)
Freq in parents	0.5633	0.4367		0.5015	0.1836		0.3117		0.0031	0.5355		0.4645	0.7562	0.2438
FBAT - Additive model														
No. of families	125		125	123		74		107	2	121		121	91	91
Z score	-0.45		0.45	1.34		1.56		-2.59	-	-1.09		1.09	1.21	-1.21
p value	0.6526		0.6526	0.1797		0.12		0.0097	-	0.2774		0.2774	0.2283	0.2283
Global stat.	chi2(1)=0.203			chi2(3)=11.733						chi2(1)=1.180			chi2(1)=1.452	
	p=0.6526			p=0.0084						p=0.2774			p=0.2283	
FBAT - Dominant model														
No. of families	70		95	82		73		94	2	79		88	32	78
Z score	-0.21		0.45	0.87		1.46		-2.47	-	-0.97		0.7	1.65	-0.52
p value	0.8316		0.6528	0.3843		0.1455		0.01443	-	0.3316		0.4838	0.09945	0.6004
Global stat.	chi2(2)=0.225			chi2(3)=7.366						chi2(2)=1.243			chi2(2)=2.85	
	p=0.8934			p=0.0611						p=0.5372			p=0.2405	
FBAT - Recessive model														
No. of families	95		70	83		18		38	0	88		79	78	32
Z score	-0.45		0.21	1.17		0.76		-1.19	-	-0.7		0.97	0.52	-1.65
p value	0.6528		0.8316	0.2438		0.4477		0.233	-	0.4838		0.3316	0.6004	0.0995
Global stat.	chi2(2)=0.225			chi2(3)=3.212						chi2(2)=1.243			chi2(2)=2.85	
	p=0.8934			p=0.3601						p=0.5372			p=0.2405	

**Marker**	**NGA19**									**rs2421853**			**rs235858**	
Allele	1 (11)		3 (13)	4 (14)		5 (15)		6 (16)	7 (17)	1 (C)		2 (T)	1 (T)	2 (C)
Freq in parents	0.0015		0.2176	0.0077		0.7114		0.0602	0.0015	0.7299		0.2701	0.6003	0.3997
FBAT - Additive model														
No. of families	1		96	5		115		32	1	102		102	109	109
Z score	-		-3.03	-		2.1		1.13	-	-3.32		3.32	4.6	-4.6
p value	-		0.0024	-		0.0353		0.2579	-	0.0009		0.0009	0.000004	0.000004
Global stat.	chi2(3)=10.162									chi2(1)=11			chi2(1)=21.146	
	p=0.0172									p=0.0009			p=4.0E-6	
FBAT - Dominant model														
No. of families	1		91	5		42		32	1	26	95		54	87
Z score	-		-2.28	-		2.83		0.93	-	-1.8	2.97		2.92	-3.9
p value	-		0.0224	-		0.0047		0.3519	-	0.0725	0.003		0.0035	0.000096
Global stat.	chi2(3)=12.711									chi2(2)=11.011			chi2(2)=21.165	
	p=0.0053									p=0.0041			p=2.5E-5	
FBAT - Recessive model														
No. of families	0		28	0		107		1	0	95		26	87	54
Z score	-		-2.53	-		0.92		-	-	-2.97		1.8	3.9	-2.92
p value	-		0.0113	-		0.3576		-	-	0.003		0.0725	0.000096	0.0035
Global stat.	chi2(2)=6.837									chi2(2)=11.011			chi2(2)=21.165	
	p=0.0328									p=0.0041			p=2.5E-5	

The polymorphisms are shown in order from the 5' end to the 3' end of the *MYOC* gene: the first four markers in the top part and the last three markers in the bottom part (See Figure 1 for details). For single nucleotide polymorphisms, the major allele was designated as 1 and minor allele as 2, with the identities of the alleles shown within brackets. For microsatellites, four alleles were found for NGA17 and six alleles for NGA19, with the number of GT repeats shown within brackets. "No. of families" refers to the number of informative families in which there was at least one heterozygous parent while "-" indicates that the corresponding value was not calculated because the number of informative families was less than 10. The degree of freedom (n) is shown within brackets after chi2: χ^2^(n), where n=1, 2, or 3.

**Figure 2:**
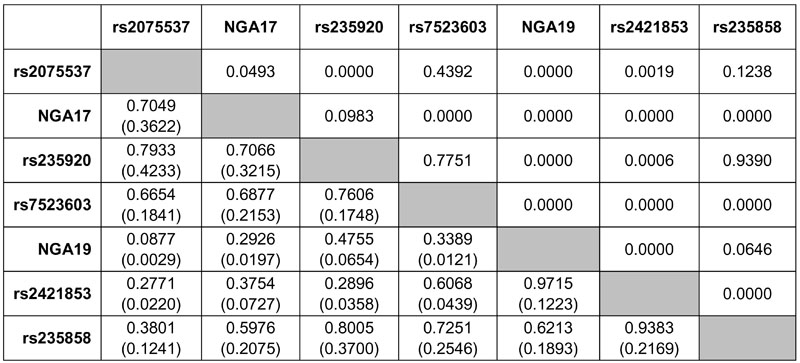
Pairwise measures of linkage disequilibrium (D' and r^2^) for *MYOC* markers under study. The cells below the diagonally descending shaded boxes show the D' (top) and r^2^ (bottom and in brackets) values. The cells above the shaded boxes show the exact p values for the corresponding linkage disequilibrium measurements.

The pairwise LD values (D' and r^2^) among these markers are shown in Figure 2. The highest two D' values were 0.9715 for the rs2421853-NGA19 pair, and 0.9383 for the pair rs2421853-rs235858 although their corresponding r^2^ values were quite small (0.1223 and 0.2169, respectively). The third highest D' value was 0.8005 for the pair rs235920-rs235858, but was not significantly different from zero (p=0.9390). The highest r^2^ value was 0.4233 for the pair rs2075537-rs235920, and the corresponding D' value was 0.7933. The LD between the microsatellite pair NGA17-NGA19 was weak (D'=0.2926 and r^2^=0.0197). In general, the LD between all other pairs of markers was weak to moderate.

### Family-based Association Test analysis

The significance level was set at 0.0219 for FBAT analysis of seven markers after correction for multiple comparisons by FDR at a level of 0.05. With this cut-off, the alternative hypothesis of showing both linkage and association was supported for four genetic markers (NGA17, NGA19, rs2421853 and rs235858) under one to three genetic models, while the null hypothesis of no linkage and no association could not be rejected for the other three markers (rs2075537, rs235920, and rs7523603) under all three genetic models tested (Table 3).

In general, preferential nontransmission (negative Z scores) was seen in allele 5 of NGA17 and allele 3 of NGA19 while preferential transmission (positive Z scores) was noted in the other alleles (Table 3). For NGA17, allele 5 showed significantly reduced transmission to the myopic offspring under additive genetic model (Z=-2.59, p=0.0097), and the global statistic was also significant (p=0.0084). Linkage and association were not apparent under dominant or recessive models. Yet, NGA19 was strongly associated with high myopia under both additive (p=0.0172) and dominant (p=0.0053) genetic models while the recessive model was not supported (p=0.0328) at FDR-adjusted significance level. It was particularly striking that allele 3 exhibited significantly reduced transmission to the myopic offspring (additive model, Z=-3.02, p=0.0024; dominant model, Z=-2.28, p=0.0224). In contrast, allele 5 showed significantly increased transmission under the additive (additive model, Z=2.10, p=0.0353; and dominant model, Z=2.83, p=0.0047). Note that allele 5 was the most common allele for NGA19 in the Chinese population under study.

At the 3' flanking region of the *MYOC* gene and also 3' to NGA19 (Figure 1), both rs2421853 and rs235858 showed significant association under all three genetic models tested (Table 3). The two alleles of each SNP showed opposite preferential transmission/nontransmission under the additive model: The Z scores were +3.32 or -3.32 for rs2421853 (p=0.0009), and +4.60 or -4.60 for rs235858 (p=4.0E-6). For rs2421853, the T allele (minor allele) exhibited significantly increased transmission under the dominant model (Z=2.97, p=0.0030), whereas the C allele (major allele) showed significantly reduced transmission under the recessive model (Z=-2.97, p=0.0030). For rs235858, the T allele (major allele) demonstrated significantly increased transmission (Z=2.92, p=0.0035) and the C allele (minor allele) significantly reduced transmission (Z=-3.90, p=9.6E-5) under the dominant model. The two alleles showed reverse preferential transmission/nontransmission under the recessive model. It is interesting to note the reciprocal relationship for dominant and recessive models when the marker is biallelic. The global statistic was also significant under the dominant/recessive models: p=0.0041 for rs2421853 and p=2.5E-5 for rs235858.

### Generation and analysis of case-pseudocontrol dataset by GenAssoc

The GenAssoc package was used to generate case and pseudocontrol subjects for CLR analysis. The dataset was first analyzed by a stepwise regression procedure. Given the main effect of NGA17, only the SNPs rs2421853 and rs235858 still contributed significant effects (p=0.0035 and 0.0001, respectively) on high myopia but not NGA19 (Table 4). Similarly, given the main effect of NGA19, only the SNPs rs2421853 and rs235858 still contributed significant effects (p=0.0063 and 1.2^-5^, respectively), but not NGA17. Given the main effect of rs2421853, only rs235858 remained significant (p=0.0001), but not NGA17 and NGA19. Similarly, given the main effect of rs235858, only rs2421853 remained significant (p=0.0415), but not NGA17 and NGA19. Finally, additional markers did not contribute significant effects to the main effects of both SNPs (rs2421853 and rs245858). In other words, this stepwise regression analysis showed that these two SNPs contributed separate significant main effects to high myopia and could explain the positive association results of the microsatellite markers.

For rs2421853 with the more frequent homozygote C/C as the reference genotype, both heterozygotes and the less frequent homozygotes were at significantly higher risk (GRR=1.678, p=0.0014 for C/T and GRR=2.738, p=0.0057 for T/T) for myopia, and the global Wald test was significant (p=9.0E-4; Table 5). For rs235858 with the less frequent homozygote C/C as the reference genotype, both heterozygotes and the more frequent homozygotes were also at significantly higher risk (GRR=2.083, p=0.0017 for C/T and GRR=3.931, p=5.5E-7 for T/T); the global Wald test was significant (p=2.0E-6). A GRR of greater than one was consistent with the increased transmission of the T allele of both SNPs under the additive model in the FBAT analysis (Table 3).

**Table 5:** Estimates of genotype relative risks based on case-pseudocontrol data using the GenAssoc package.

**Marker**	**Genotype**	**GRR**	**Z score**	**p value**	**95% CI**	**Global Wald test p value**
rs2421853	C/C	1	-	-	-	9.00E-04
	C/T	1.678	3.19	0.0014	1.222-2.308	
	T/T	2.738	2.76	0.0057	1.340-5.593	

rs235858	C/C	1	-	-	-	2.00E-06
	C/T	2.083	3.13	0.0017	1.316-3.298	
	T/T	3.931	5.01	5.50E-07	2.301-6.717	

The table shows the effect sizes of the genotypes in terms of genotype relative risk (GRR) and the corresponding 95% confidence intervals (CI). For each genotype, the Z score is the statistic testing the deviation of the GRR from 1 and the significance level as the p value. For each SNP, the significance of the global Wald test is indicated in the last column. Robust variance estimates were used to account for correlation between contributions from nuclear families with two or more siblings.

## Discussion

In a population-based case-control study involving 97 high myopes (-8.00D or less) and 92 matched emmetropes of Chinese ethnicity in Singapore [36], researchers found significant allelic association between NGA17 and myopia and observed the relative risk for myopia increased from 0.7 to 4.3 as the repeat length decreased. In the present family-based study, the long allele 5 was indeed found protective (Z=-2.59; Table 3), but the increasing trend of relative risk with decreasing repeat length was not observed. Interestingly, association between myopia and NGA19 was not found in this Singapore study, but was demonstrated in a second Singapore study [37], which will be described in the next paragraph, and in the present study (Table 3). When a reanalysis was performed for a subset of our families (n=74) with siblings (n=95) having more severe myopia (-8.00D or less; this is the same threshold used in the Singapore case-control study), no association could be demonstrated between *MYOC* microsatellites (both NGA17 and NGA19) and myopia under any genetic models tested (data not shown). This discrepancy remains to be explained, although one possible reason might be the different distribution of refractive errors in the two groups of subjects.

The Singapore group later replicated their own initial case-control study with an independent sample set of 104 Chinese families each with at least one severely myopic child [37], but the threshold of refractive error for inclusion was not clearly indicated. Significant association (p=0.0014) of NGA19 with myopia was demonstrated with increased transmission of allele 128, which was very likely the same as our allele 5 on the basis of similar allele frequencies (0.679 versus 0.711). This is consistent with the significantly increased transmission of allele 5 under additive (Z=2.1) and dominant (Z=2.83) models (Table 3). However, this Singapore study [37] did not find any association with NGA17, which was found to be associated with high myopia in their first study [36] and in the present study (Table 3).

Another group conducted a case-control study involving 70 high myopes (-6.00D or less) and 69 adult nonmyopic controls of Chinese ethnicity. In this study, only the promoter microsatellite NGA17 was tested [38]. No association was found (p=0.84). The NGA17 allele frequencies reported by Leung et al. [38] were similar to those reported in the present study (p=0.7307). The same threshold of refractive error (-6.00D or less) was used in these two studies involving Chinese subjects in Hong Kong. The threshold of -6.00D or less was commonly used to define high myopia [11-13,15]. A reanalysis carried out using only one myopic (-6.00D or less) child from 70 families of our collection did not demonstrate association between NGA17 and high myopia under the three genetic models tested. It is thus probable that the nonreplication in the study conducted by Leung et al [38] might be an issue of sample size, although other possibilities cannot be ruled out. It is also interesting to note that the second Singapore study did not demonstrate association with NGA17 either [37].

All four genetic association studies were done in Chinese populations, but they differed in several aspects: study design (family-based or population-based), sample size, and the cut-off value of refractive error for inclusion in the study. The present study is the largest of these four to test the association between *MYOC* markers and high myopia in that it involved a total of 162 families with 233 severely myopic offspring. It indicates linkage and association of NGA17 with high myopia with significantly reduced transmission of allele 5 (with 15 GT repeats) to myopic offspring under an additive model (Table 3). It also demonstrates strong linkage and association of NGA19 with high myopia with significantly reduced transmission of allele 3 (13 GT repeats) under all three models tested (Table 3). These results support the preliminary findings from Singapore.

The present study is a family-based association study and hence the positive association results are robust to problems due to population admixture or stratification [39,49]. Thus, the positive association can be due to either the direct effect of the marker tested or the marker tested being in LD with an unknown causative sequence variation in the nearby region.

The microsatellites tested might not be the causative sequence variations accounting directly for the association with myopia, although it has been suggested microsatellites play important roles in the regulation of transcription and potential roles in complex traits and diseases [50]. Instead, the tested microsatellites might be in LD with the genuine causative SNPs within or around the *MYOC* locus. We initially used a panel of 40 unrelated Chinese subjects to screen for SNPs in the three *MYOC* exons, and we found four coding SNPs (rs2234926 and rs2234927, and two others not reported in public databases). However, their minor allele frequencies were each discovered to be less than 5% among 150 parents from our collected families (data not shown). Thus, they were not ideal for association studies. SNPs in noncoding regions were then used to further delineate the relationship between *MYOC* and high myopia.

Five tag SNPs were selected based on the Han Chinese genotype data of the HapMap database (Figure 1). These SNPs spread across the *MYOC* gene. As expected, there were no significant differences in their allele frequencies (p values: 0.0610-0.9083) and genotype frequencies (p values: 0.1464-0.9937) between the Han Chinese group (n=45) in the HapMap database and the Chinese parental group (n=324) of this study (data not shown). Three SNPs (rs2075537 and rs235920 from the 5' flanking region, and rs7523603 from intron 1) did not show any evidence of linkage and association with high myopia (Table 3). On the contrary, two other SNPs (rs2421853 and rs235858) showed strong evidence of linkage and association (because of low p values) with high myopia (Table 3). This is the first report of a novel finding. Note that rs2421853 is about 4.5 kb and rs235858 about 7.9 kb downstream of the microsatellite NGA19 in the 3' flanking region.

FBAT analysis thus showed the linkage and association of two microsatellites and two SNPs with high myopia (Table 3). A stepwise CLR analysis of the case-pseudocontrol dataset demonstrated that the two SNPs at the 3' flanking region had separate main effects on high myopia and could each account for the association of NGA17 or NGA19 with high myopia (Table 4). This finding may explain the apparently discrepant association results of the microsatellites reported in the two Singapore studies mentioned [35,36]. Moreover, it suggests that these two 3' SNPs may have separate functional effects or are in LD with other separate functional polymorphisms. GRR estimates were compatible with an additive model for both SNPs: GRR increased from 1.678 to 2.738 and from 2.083 to 3.931 when the copy number of the risk allele A was increased from one to two for rs2421853 and rs235858, respectively (Table 5). This was consistent with the finding that, of the three models tested, the additive model gave the lowest p values in FBAT analysis (Table 3).

**Table 4:** Wald test of main effects in a forward stepwise regression analysis of case-pseudocontrol dataset.

		**Wald test statistic**		
**Null model**	**Alternative model**	**chi2**	**df**	**P**
NGA17	NGA17 + NGA19	4.79	2	0.0914
NGA17	NGA17 + rs2421853	11.33	2	0.0035
NGA17	NGA17 + rs235858	18.43	2	0.0001

NGA19	NGA19 + NGA17	5.3	2	0.0706
NGA19	NGA19 + rs2421853	10.13	2	0.0063
NGA19	NGA19 + rs235858	22.61	2	1.20E-05

rs2421853	rs2421853 + NGA17	3.64	2	0.1622
rs2421853	rs2421853 + NGA19	3.86	2	0.1455
rs2421853	rs2421853 + rs235858	17.88	2	0.0001

rs235858	rs235858 + NGA17	0.52	2	0.771
rs235858	rs235858 + NGA19	4	2	0.1355
rs235858	rs235858 + rs2421853	6.36	2	0.0415

rs2421853 + rs235858	rs2421853 + rs235858 + NGA17	0.79	2	0.674
rs2421853 + rs235858	rs2421853 + rs235858 + NGA19	4.11	2	0.1283
rs2421853 + rs235858	rs2421853 + rs235858 + NGA17 + NGA19	5.08	2	0.2789

For NGA17 and NGA19, the case-pseudocontrol dataset was generated after grouping all non-5 alleles into one allele.

With haplotypes generated by expectation-maximization algorithm [43,44], haplotype analysis by FBAT indicated that the p values of global statistics became less extreme, though still significant after correction for multiple testing, whenever one or more marker (rs2421853, NGA17 or NGA19) was added to the rs235858-carrying haplotypes (data not shown). In other words, the association of rs235858 with high myopia gave the lowest p values on its own under all three genetic models. Given the main effect of rs235858, the contribution of rs2421853 was still significant (Table 4). The p value of the Wald test (0.0415) was close to 0.05. SNP rs2421853 is about 3.5 kb apart from and in strong LD (D'=0.9383) with rs235858 (Figure 1 and Figure 2). However, given the same sample size, their low pairwise r^2^ value (0.2169; Figure 2) implies that rs2421853 can only partially capture the genetic information of rs235858 [51]. Therefore, it is possible that rs235858 may also account for the association of rs2421853 with high myopia.

Sequence analysis of rs235858 and its flanking regions by the online software SIGNAL SCAN revealed interesting results. The short sequence C(A)TCTG indicates the T allele of rs235858 (shown within brackets) and its flanking sequences in the antisense strand. This matches the consensus recognition sequence CANNTG for transcription factors of the helix-loop-helix type [52]. In addition, the sequence CATCTG was found to be one of the three sequence motifs for a 3' enhancer located 12 kb downstream of the human immunoglobulin kappa gene [53]. Site-directed mutagenesis and electrophoretic mobility shift assay indicated that distal pairs of dinucleotides in the (CA)TC(TG) sequence were critical to the in vitro enhancer activity [53]. Whether the sequence CATCTG forms part of the motifs for any enhancer downstream of the *MYOC* gene remains to be determined and is worthy of further investigation. It is tempting to speculate that the C allele sequence context C(G)TCTG might reduce the activity of any potential enhancer to be found.

On the other hand, the significant results of rs2421853 and rs235858 may be due to their strong LD (high D' and r^2^) with other neighboring SNPs. We suggest that further investigation be performed for these neighboring SNPs in the 3' flanking region of the *MYOC* gene. The most updated HapMap data (release 21, phase II, July, 2006) indicate that the *MYOC* gene is bounded within a 60 kb region by two recombination hotspots and hence separated from other flanking genes by these hotspots. This makes it unlikely that any causative *MYOC* SNP associated with high myopia would fall outside of this 60 kb region.

Interestingly, a few large studies of genome-wide linkage analysis for myopia families did not show linkage to the *MYOC* locus [13,16,19,23]. Locus heterogeneity underlying different Mendelian forms of high myopia in different families is one possible reason [25]. Alternatively, the power of a linkage analysis study depends on the density of genetic markers genotyped, the effect size of the locus (or the GRR), and the sample size (or the number of informative meioses) [25]. Several studies genotyped microsatellites at a density of about 10 cM intervals [13,16,19], which is not dense enough for complex diseases with low to moderate GRRs. The present study showed a moderate GRR of about 2-4 for rs235858 (Table 5). Another study used a marker set with a higher density of 5 cM intervals, but the study subjects had refractive errors spreading from hyperopia to myopia with a mean of +0.31 D [23]. Indeed, these findings signify that linkage analysis is not powerful enough for mapping loci showing small genetic effects [21,22,24].

In summary, two microsatellites and two SNPs were found by FBAT analysis to show linkage and association with high myopia in the Hong Kong Chinese population under study. A stepwise CLR analysis of case-pseudocontrol dataset indicated that the two SNPs could each account for the association results of the two microsatellites and that these two SNPs seemed to exhibit separate main effects on high myopia. The high D' yet low r^2^ values between these two SNPs suggested that the significant association of rs2421853 might also be accounted for by rs235858, whose impressively low p values in FBAT (4.0E-6) and GenAssoc (4.0E-6) analyses gave us high degree of confidence in the positive association results. These are novel findings never reported before and thus should be replicated using independent sample sets, preferably from populations of different ethnic origins (e.g., Caucasian) [54]. Top priority should be given to SNPs in the 3' flanking region of the *MYOC* gene, particularly those that may have regulatory functions modulating gene expression. Such SNPs should then be further studied to confirm their putative regulatory functions experimentally.
